# Exploring Well-being at Work—An Interview Study on How IT Professionals Perceive Their Workplace

**DOI:** 10.3389/fpsyg.2021.688219

**Published:** 2021-08-02

**Authors:** Stefanie Zutavern, Jürgen Seifried

**Affiliations:** Economic and Business Education – Professional Teaching and Learning, University of Mannheim, Mannheim, Germany

**Keywords:** well-being at work, perception, work environment, commitment, IT professionals, interview study

## Abstract

The workplace is particularly important for promoting well-being at work and general life satisfaction, as performing a professional activity can be perceived as satisfying and motivating. In addition, employment opens up opportunities for individual development that employees may be perceived as fulfilling. By conducting an interview study with IT professionals of a German medium-sized company, we investigate which factors of the individual work environment are perceived as conducive to the performance of everyday job duties and thus increase well-being at work. Furthermore, we analyze the extent to which participants are satisfied with the implementation of the factors that are important to them, whether socio-demographic differences are relevant, and whether the perception of the work environment has an effect on employees' commitment. Results show that interpersonal factors in particular are considered to be important in everyday working life. About individual factors, a mixed picture emerged, whereby sociodemographic differences play only a minor role. Furthermore, there are indications of a positive relationship between the perception of the work environment and the IT professionals' commitment. In-depth analysis of the employee statements helps to determine which aspects of the work environment should be implemented, developed, or promoted. In the long term, this can support individual learning and development paths and generates a work environment that sustainably promotes employees' well-being at work and fosters long-term employment relationships.

## Introduction

For most people, work is a prominent part of their lives. Not at least because they spend a large proportion of their available time at the workplace. It is therefore important that employees feel good in their work environment. This state is achieved when employees have a positive perception of their work environment (Buffet et al., 2013). More specifically, well-being at work is an individual's assessment of his or her work environment, in which the individual positively evaluates the conditions that shape the respective work environment. A high level of satisfaction with workplace conditions is meaningful because it can have several positive effects on the individual. For instance, studies show that full-time employees rate their life satisfaction as better than the unemployed (Eiffe et al., 2016; Mousteri et al., 2018). Moreover, high subjective well-being has a positive impact on health and life expectancy (DeNeve et al., 2013). Happy and satisfied individuals also benefit in terms of interpersonal relationships, as they are particularly collaborative and cooperative (DeNeve et al., 2013). Furthermore, happy employees seem to be more productive in comparison to their unhappy counterparts (Peiró et al., 2019).

Given that barely one-third of well-being is attributed to genetic predispositions, nearly two-thirds of well-being can be caused by environmental influences (Diener et al., 2018). Thus, workplace interventions can help to improve employees' well-being. Factors from the work environment that are potentially conducive to influence well-being can be grouped into three categories (empirical findings on the effects of the respective factors are presented in the section on conducive factors to well-being at work): Category 1 concerns factors of the social environment. It includes interpersonal relationships in the work context, characteristics of the work climate, opportunities for internal and external collaboration, and employees' relationships with colleagues and supervisors. Category 2 addresses work characteristics. In addition to the opportunity to take on responsibility and act independently, the relevance of the work and its holistic nature as well as the variety of tasks and feedback also play a role. In addition, the psychological, cognitive, and quantitative requirements of the work and general working conditions are crucial for promoting employees' well-being. Finally, employer characteristics are of relevance (Category 3). These cover aspects of work organization, such as processes and information flow, as well as culture-related topics, such as opportunities for professional and personal development, work-life balance programs, and leadership style. Whether or not employees perceive their work context positively depends largely on how they experience and assess the factors that shape their work environment (Fisher, 2010). Accordingly, employees may benefit in different ways from the available resources of their workplace (Louws et al., 2016). Employers should therefore find out which factors are relevant from the employee's point of view. By aligning the work environment with the needs of their employees, it is more likely that employees will feel comfortable in the respective work environment.

Especially in times of a shortage of skilled workers, employers need to be perceived as attractive. This is especially true for the IT sector. Since IT specialists are currently in high demand, it is particularly easy for them to change employers. Mainly large employers offer extensive benefits to make themselves attractive to skilled personnel. Medium-sized employers—who usually have fewer resources at their disposal—are therefore faced with the challenge of retaining their employees. By creating a work environment that is satisfactory from the employee's point of view, it is possible to successfully stand out from the competition and achieve long-term personnel ties.

Against this background, we want to learn more about how the employees of a medium-sized IT service provider in the financial sector perceive their work environment. We investigate which aspects IT professionals perceive as conducive to the performance of their everyday work since employees' perception influences the use and application of (learning) resources (Hoekstra et al., 2009; Louws et al., 2016). Using semi-structured interviews, we provide in-depth insights into the employees' perception of their work environment and contribute to better understand how employee perceptions can lead to satisfaction and well-being at work. This understanding can help sustain employees' well-being at work and overall life satisfaction in the long run.

In the following section, we present the theoretical background and address the underlying empirical findings as well as our research model. This is followed by a description of the methodology and data sample. Next, we outline the results of the semi-structured interviews and classify them in the existing literature. The paper closes with a discussion of the limitations and scientific significance of the study.

## On The Relationship Between One's Workplace and Well-Being At Work

### Conceptualizing Well-being

By now, researchers from different disciplines (e.g., psychology, sociology, or economics) have turned their attention to the construct of well-being. Together, they share a common understanding of well-being, which can be characterized by the following three features: First, well-being is the result of a global judgement and its degree expresses an overall evaluation of life (Wright and Bonett, 2007; Diener et al., 2017). Second, affect and emotion play a role in assessing one's life. This indicates, that well-being is strongly influenced by how individuals perceive their environment (Bowling et al., 2010; Diener et al., 2017). Third, an individual's global judgement is influenced by various factors, which can be assigned to different life domains (Eiffe et al., 2016). Such domains are, for instance, one's living conditions, health, and education. Also, the work environment represents a subdomain that influences an individual's well-being evaluation. Taken together, an individual's well-being results from the subjective overall assessment of various domains of life. This understanding of well-being is referred to as part-whole theory and is based on findings demonstrating the link between job satisfaction and well-being (recently confirmed by Cannas et al., 2019, for an overview and comparison to other theoretical approaches, see, e.g., Bowling et al., 2010). Following the part-whole theory, there is a hierarchical order between one's satisfaction with a specific area of life and overall well-being, which comprises a total of three levels. Thereby, overall well-being forms the highest level. The middle level is composed of the satisfaction scores for various life domain. Finally, the third level comprises the evaluation of all factors that make up this particular life domain. Considered in summary, the part-whole theory is based on a very broad conceptual understanding of well-being, according to which many different factors plus their perception needs to be taken into account (details are explained in the section on the relevance of employee perceptions).

Within the scope of our research project, we follow the part-whole theory and focus on the subdomain work. In this context, well-being is often specified as well-being at work or well-being at the workplace, which is about creating a work environment that is perceived as positive by employees (Buffet et al., 2013). More specifically, it is about enabling “safe, healthy and productive work in a well-led organization by competent workers and work communities who find their job meaningful and rewarding and see work as a factor that supports their life management” (Buffet et al., 2013, p. 14). In this way, employees should be allowed to unfold their potential in the best possible way (Schulte and Vainio, 2010) to reach an “optimal psychological functioning and experience at work” (Gruman and Saks, 2013). Based on these arguments, it becomes clear that well-being at work is an individual assessment of the work environment, which depends on the subjective perception of the conditions forming this setting.

Hence, by focusing on the design of the work environment employers can positively influence the well-being of their employees in two respects. On the one hand, this “conditional approach” (Pot, 2017, p. 96) aims at preventive action. Accordingly, all features of the work environment should be designed in such a way that they promote well-being at work (primary prevention, Pot, 2017). This means that the factors which positively influence employees' well-being are specifically promoted and, at the same time, potentially negative influencing factors are reduced. Implementing such an approach can primarily reduce employee strain by protecting employees from the consequences of low well-being at work (e.g., exhaustion, inefficiency, and stress as consequences of burnout; Patel et al., 2018). Moreover, if primary prevention succeeds, initiatives to support employees in coping with low well-being (secondary prevention, Patel et al., 2018) become obsolete. On the other hand, examining the work environment can lay the foundation for the sustainable development of an organization and its employees. This perspective is introduced as the psychology of sustainability and sustainable development by Di Fabio (2017). The aim here is to implement the reflection of prevailing working conditions as a fixed process so that design potentials for a work environment conducive to well-being at work can be derived continuously. Referring to the part-whole theory, it, therefore, seems a promising starting point for organizations to focus on factors shaping the work environment—and thus the lowest of the three levels—to promote employees' well-being at work.

### Conducive Factors to Well-being at Work

Research shows that a variety of factors influence well-being at work. Concerning this connection, we have conducted literature research and identified a total of 24 factors, which—roughly speaking—can be divided into three categories, namely aspects related to either the social environment, work characteristics, or employer characteristics. The category *social environment* refers to interpersonal relationships in the work context and comprises work climate characteristics, opportunities for internal and external cooperation, as well as employees' relationship with colleagues and supervisors. In this respect, positive influences such as social inclusion and support as well as negative influences such as bullying or discrimination play a role. For instance, work climate characteristics such as the feeling of being understood and accepted in the team, as well as social support, help employees cope with stress and heavy workload (Aalto et al., 2018), decrease the risk of burnout and foster job satisfaction (Van der Heijden et al., 2020). Moreover, work engagement is positively influenced by a collaborative and constructive team climate (Albrecht, 2012), which also reduces bullying (Olsen et al., 2017). A workplace free of bullying in turn promotes job satisfaction (Olsen et al., 2017) and reduces the risk of burnout (Steffgen et al., 2020). Also, the opportunity of making friends at work has a positive effect on job satisfaction (Morgeson and Humphrey, 2006). While these findings apply to relationships at the same hierarchical level and within the organization, other studies proved that relationships with supervisors (Chang and Cheng, 2014) as well as interaction with external cooperation partners (Morgeson and Humphrey, 2006) also affect employee job satisfaction.

Concerning *work characteristics*, studies pointed out their positive influence on employees' satisfaction (e.g., Hackman and Oldham, 1974) and well-being (e.g., Karasek, 1979; Siegrist, 1996; Bakker and Demerouti, 2007) since decades. In this regard, characteristics of the work tasks, as well as requirements associated with the occupational activity and technical-organizational framework conditions to fulfill one's job duties, are decisive. Motivational design parameters such as autonomy or participation in decision-making processes can have a favorable effect on employee engagement (Albrecht, 2012) as well as on employees' job satisfaction and commitment (Uribetxebarria et al., 2020). The same applies to the meaningfulness of one's work tasks (Van der Heijden et al., 2020) as well as their variety and feedback through work (Morgeson and Humphrey, 2006). In addition, basic conditions for performing the job, such as available technologies and equipment or room temperature and spatial design, can have a positive effect (Morgeson and Humphrey, 2006). However, the work environment is considered unfavorable when psychological, physical, and quantitative demands become excessive from the employee's point of view, causing burnout and physical problems in the worst case (Van der Heijden et al., 2020; Bianchi et al., 2021). In summary, responsibility and autonomy, the significance of the work and its holistic nature, task variety and feedback on the job, in addition to psychological, cognitive, and quantitative demands and general working conditions, are decisive work characteristics for promoting well-being at work.

Finally, characteristics that have an organization-wide impact can also affect employees' well-being. About *employer characteristics* that apply across departments and activities, cultural, and work organization aspects are particularly important. For instance, an organizational culture defined by openness, fairness, and support has a positive impact on employees' engagement, commitment, and extra-role behavior (Albrecht, 2012). Furthermore, the health awareness of supervisors plays a role in employees' well-being, as it is reflected in their leadership style and can positively condition employees' mental health (i.e., depression and anxiety symptoms; Vonderlin et al., 2021). In addition, employees seem to be more proud, motivated, and overall satisfied when their employer has a positive reputation (Tanwar and Prasad, 2016). Similarly, development opportunities promote job satisfaction and commitment to the employer (Uribetxebarria et al., 2020) and have a positive impact on subjective well-being (Eiffe et al., 2016). Increased well-being could also be linked to informal learning activities in the workplace (Jenkins and Mostafa, 2015; Jeong et al., 2018). At the same time, opportunities to acquire new skills and knowledge reduce the risk of burnout (Bianchi et al., 2021). In contrast, burnout is promoted when family and work are difficult to reconcile (work-life conflict, Steffgen et al., 2020; Bianchi et al., 2021). Stress is also increased when employees perceive their job or specific job features as being at risk (quantitative and qualitative job insecurity, Chirumbolo et al., 2017). In addition to cultural aspects, employee satisfaction is also conditioned by work organizational aspects. For example, a well-functioning information flow provides access to information, resources, and mutual support, as well as development and learning opportunities. All these features shape an environment in which knowledge is shared. This fosters individual skill development and increases satisfaction (Trivellas et al., 2015). Finally, internal and external process quality also plays a role. Smooth and efficient work processes make it easier for employees to perform their tasks. This reduces the workload and makes employees more satisfied with their job (Chiang and Wu, 2014).

### Well-being and Commitment

If an employer succeeds in creating a work environment in which its employees feel good, both parties can achieve further positive effects. For example, research suggests that high levels of well-being and job satisfaction are associated with an increased commitment to the employer (Jain et al., 2009; Aggarwal-Gupta et al., 2010; Culibrk et al., 2018) and employees with high levels of commitment show lower turnover intention (Agarwal and Sajid, 2017). This is positive from an employee's perspective in that it avoids the negative consequences of changing employers for those who stay with their current organization. On the one hand, these can be monetary burdens, such as application or relocation costs. On the other hand, a change of employer can have negative psychological consequences, e.g., social pressure caused by integration efforts in the new work environment or stress that can arise with the emerging intention to quit. The employer also benefits from highly committed employees. With low turnover, there are no direct costs for replacement, training the new hire, or productivity losses. At the same time, indirect costs are avoided that can arise from spill-over effects on other employees or declining motivation among the remaining workforce (O'Connell and Kung, 2007; Kuhn and Yu, 2021). All in all, staying with the current employer allows avoiding unpleasant consequences while maintaining a positive state of high well-being and commitment. To support long-lasting employment relationships, studies point to the need to focus on the organizational context and how it is perceived by employees, as this is significant for retention (Koslowsky et al., 2012) and organizational commitment (Herrera and De Las Heras-Rosas, 2021).

### The Relevance of Employee Perception

By defining well-being at work as an individual's assessment of the work environment depending on the subjective perception of the conditions forming this work environment, we have emphasized that employee perception plays a crucial role in promoting well-being at work. As Fisher (2010) notes “it is important to remember that happiness and positive attitudes are not directly created by environments or events […], but rather by individuals' perceptions, interpretations, and appraisals of those environments and events” (p. 396f.). This implies that employees of the same organization do not necessarily benefit equally from the prevailing working conditions, because they perceive available resources of their work in different ways (Louws et al., 2016).

The perception of environmental conditions is an important field of research in different diciplines (e.g., artificial intelligence, robotics, marketing, pedagogic, or psychology). For the question addressed in this paper, it is worthwhile to take a closer look at psychological research. For example, researchers from environmental psychology, a subdiscipline of industrial and organizational psychology, are addressing the relevance of perception. The focus is on the interaction between the environment and the individual, and the work environment is one among many fields of research (Bell and Sundstrom, 1997; DeYoung, 2013). To analyze the interactions between the environment and the individual, environmental psychology takes a holistic approach that aims to gain insights into factors that influence human behavior and well-being (DeYoung, 1999, 2013). In relation to well-being at work, such insights can help to identify drivers of well-being in the workplace. By considering these insights, work environments can be designed to best meet the needs of their employees. Other psychological approaches also support the finding that perceptual processes are of paramount importance. The interaction of individual factors and environmental factors as well as their perception is also analyzed within the framework of the theory of action regulation (Hacker, 1973, 2003, 2020; Volpert, 1983). It is assumed that the execution of an activity is conditioned by environmental and individual factors, and the perception of the employees is considered crucial for the processing of the environmental factors. Environmental factors are, for example, economic, social, work-organizational, or technical conditions that unfold within organizational structures and can give employees leeway to regulate their activities (Hacker, 2020). Individual factors refer to factors that employees bring to the work environment. These include physical prerequisites as well as education, cognitive abilities, and motivational aspects (Hacker, 2020). Finally, psychological processes (perception, thinking, remembering, motivation, emotion, and volition), representations of memory (mental models including norms and goals used to guide future actions), and psychological characteristics (especially competencies) of employees are crucial to the process of action regulation. In the context of well-being at work, action regulation theory illustrates that processing influences from the work environment start with employees' perceptions.

Studies on employees' perceptions of learning opportunities at the workplace underline the relevance of individual perceptions in assessing the work environment. For instance, Hoekstra et al. (2009) use the example of teachers to show that equal working conditions lead to different learning activities. While one teacher perceives the provided degree of autonomy as an opportunity for development and uses this freedom to try out different working styles, a colleague in the same school finds it a lack of guidance. The same applies to participation in reflective dialogues and feedback. One teacher perceives the context as a chance to develop one's performance and actively seeks feedback and exchange. The colleague experiences feedback as unpleasant criticism and avoids such situations and, thus, tends to stay in his or her comfort zone. More recent findings also show that it “is not so much the objective conditions that support or impede professional learning but the way teachers perceive those workplace conditions that influence teachers' learning” (Louws et al., 2016, p. 770). Once participants perceive the prevailing structural and cultural conditions positively, they are more likely to engage in continuous professional development, take on responsibility, and tend to be more self-directed (Louws et al., 2016). In contrast, perceptions of a constraining work environment can lead to focusing on task-related goals without having a broader perspective (Louws et al., 2016). Thereby, experienced support is crucial here, with colleagues, supervisors, and mentors being all relevant (Fox et al., 2010). Bryson et al. (2006) confirm this for employees of a winery. Their study indicates that access to and take-up of professional development opportunities depend on employees' managers. Van der Rijt et al. (2013) come to similar conclusions in the case of employees in various commercial departments. Although they speak more generally of expertise providers, participants report that perceived quality and access to expertise as well as trust in the expertise providers are decisive in determining whether and how often they ask for help.

At this point, it should be noted that differences in the perception of the work environment can also be explained by socio-demographic factors. For example, discrimination has a greater impact on job satisfaction among younger and older employees than among middle-aged employees (Taylor et al., 2013). While younger employees tend to find satisfaction in the significance of their tasks, older employees benefit from the opportunity to exert influence (Van der Heijden et al., 2020). In addition, the risk of burnout decreases for older employees the more support they experience at work (Van der Heijden et al., 2020).

### Research Model and Research Questions

**Figure 1:**
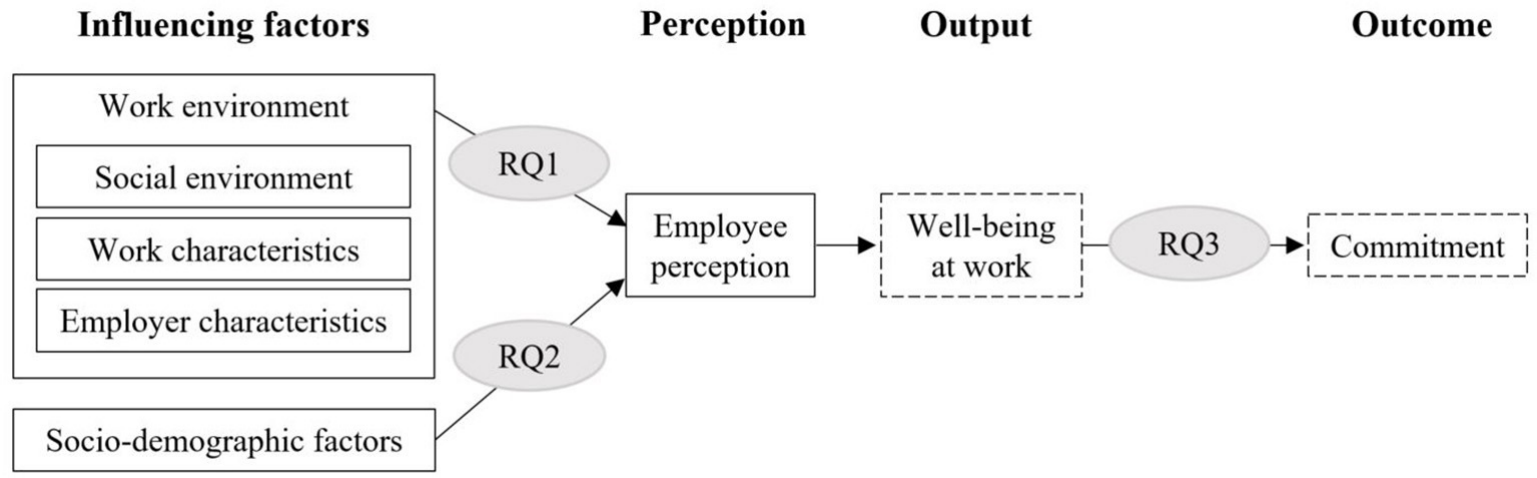
Research model.

The findings discussed regarding employees' well-being at work and their perception of the work context are consistent with the part-whole theory and our reasoning regarding factors conducive to well-being at work, emphasizing that employees' perceptions of the work environment is influenced by a variety of factors. Against this background, we have combined the outlined theoretical considerations into a research model, taking into account the empirical findings on well-being at work (see Figure 1). In addition to factors that promote well-being at work, we consider employees' perception, well-being at work, and employees' commitment. According to action regulation theory, the influencing factors are composed of environmental and individual factors. Related to our research project, these are factors conducive to well-being at work. The social environment, work characteristics, and employer characteristics together shape the work environment and are classified as environmental factors. The socio-demographic factors are classified as individual factors. Employee perceptions trigger the process of action regulation and determine the subsequent development of well-being at work (output), which affects the level of engagement (outcome).

As illustrated in the research model, the entire context in which work is performed plays a crucial role in promoting employees' well-being. As such, it is necessary to look at the work environment from a holistic perspective to determine factors influencing well-being in the workplace. With this study, we aim to do so and provide deeper insights into how employees perceive their work environment. We want to learn more about how employees experience various aspects of their work environment when they consider their work environment as a whole. Respectively, research question one addresses employees' perception of the work environment and is surveyed via two questions. First, we wanted to know:

RQ1.1: What aspects of the individual work environment do employees perceive to be conducive to carry out their work tasks?

Furthermore, we wanted to ascertain which specific circumstances lead to a positive perception of particular work environment factors. Such insights could reveal whether there are differences in the perception of the work environment within an organization and what causes them. Knowledge about different perceptions within an organization could help to identify best practice and problematic situations. Corresponding research activities such as ours could contribute to developing suggestions for designing a satisfactory work environment. To this end, we further investigated:

RQ1.2: To what extent perceive employees specific factors of the work environment as realized in their daily work context?

To account for the influence of socio-demographic factors in our study, we additionally analyzed the following question:

RQ2: Does employees' perception of factors of the work environment differ for distinct socio-demographic groups?

Finally, we investigated the extent to which employees' perceptions of the work environment are related to their commitment, asking:

RQ3: Is there a relationship between employees' perception of the work environment and their commitment to the employer?

## Method

### Context of the Study

The study was conducted with a medium-sized IT service provider operating in the financial sector in Germany. The company offers its customers IT solutions that include the development of software as well as its implementation and operation. To work in a customer-oriented manner, the employees strive to develop innovative solutions that account for the customers' needs. In doing so, the employees have to deal with frequently changing demands. New demands result from the dynamic change of the (technical) development within the industry, which causes innovations in the company's processes and products. Furthermore, customer requirements can change (at short notice), so that flexible adjustments to ongoing project work are commonplace. These conditions make frequent changes in work tasks and processes characteristic of everyday work for the consulted IT professionals. Accordingly, it is particularly important for employees to work in an environment they perceive as positive and which encourages them in the performance of their daily tasks. In this way, a contribution can be made to their well-being at work. To understand the prerequisites of building such a work environment, the present study aims to find out which specific aspects cause a positive perception of those factors shaping the consulted IT professionals' work environment.

### Instrument

In attempting to determine how the well-being of IT professionals can be supported by the design of their daily work routine, we are interested in how employees perceive certain aspects of their work context. As noted above, research has shown that employees' perception of these relationships is highly subjective. Qualitative research methods allow depicting such subjective phenomena (Yin, 2018) because they are particularly suitable for capturing individual experiences and placing them concerning the participants' reality of life (Patton, 2015). In this context, open-ended questions provide the opportunity to gain in-depth and context-related insights into the phenomena of interest (Patton, 2015; Yin, 2018). For this reason, we have decided to conduct semi-structured interviews to ask the IT professionals about aspects of their work environment, which they perceived to be conducive to carry out their job duties. We encouraged participants through six questions to provide in-depth insights into how they perceive a total of 24 factors we have identified in the literature as influencing factors (the factors were introduced in the section on the relationship between one's job and well-being at work). The first question concerned the general conditions of work, and thus covered the category employer characteristics. Questions two to six were related to work tasks, the scope of work, emotional experience, professional requirements, collaboration, and communication, and covered the category work characteristics. Aspects of the category social environment were addressed by all questions but in particular by the questions touching on emotional experience, cooperation, and communication. Each question started with a short introduction that prompted the participant to focus on the work context. Afterwards, the interviewer asked about supporting factors within that field. Due to the rather general nature of the questions, we decided to give two examples per question to guide the participants. This seemed reasonable, considering the potential range of factors and differences in individual perceptions. For example, the question relating to general conditions of work was as follows: “Please think about your workplace: Which general conditions at your workplace do you find particularly conducive to carry out your work tasks? How important are these points to you? Consider the following aspects—for example, career and development opportunities or the compatibility of work and family life.” The examples were identical for all participants, and that meant the participants' statements could be compared (Nohl, 2013). Before the next question was asked, the interviewer summarized the top three factors to which the participant attached particular importance while responding. Participants either confirmed or corrected this summary and finally weighted it. This resulted in an individual ranking of the three most important factors per question and an additional check whether the interviewer had correctly recorded the participant's answers.

Additionally to the perception of the work environment, we asked the IT professionals at the end of the interview to assess how committed they feel to their employer. The question was taken from the KUT questionnaire for assessing commitment (Klein et al., 2014) and reads, “How committed are you to your employer?” Since the question is again open-ended, we formulated two hints to help participants answer the question, as we did in the previous questions. Both hints are based on items from Mowday et al. (1979) questionnaire on organizational commitment and read “Think of statements such as the future of my employer is important to me, or I am proud to work for this employer.” We tested the instrument's comprehensibility and practicability within a pre-test (*N* = 3).

### Data Collection and Sample

To obtain the sample, the entire workforce of the IT service provider was informed via the company's intranet. For this purpose, we introduced the study briefly in an information letter. All employees were invited to participate via the information letter and it was explicitly pointed out that participation was voluntary and answers will be processed anonymously. In case the employees were interested in participating, they were asked to share their socio-demographic data via an online link presented in the information letter. Thereby, we aimed to recruit a sample that best represents the IT service provider's workforce. Additionally, the IT professionals were asked to share their contact details via this link so that we could contact them to arrange an interview appointment.

The final sample (*N* = 61, see Table 1) was drawn from 89 valid responses, representing a response rate of 23%. The majority of the participants were male (74%), which reflects the actual gender distribution in the company. Employees aged 30 or younger (18%) were over-represented, while older employees (51–60 years) were under-represented (33%). Nevertheless, the total sample shows a relatively balanced distribution across the age groups. More than half of the participants had 21 or more years of professional experience (59%) and had been working for this employer for more than 10 years (55%). In total, the sample represents all organizational units and all three locations of the IT service provider. Due to the high proportion of younger participants, of whom 73% were in a qualification phase, trainees and students (training and development) were overrepresented with 14%.

**Table 1:** Sample (all figures in percent, *N* = 61).

**Criterion**	**Expression**	**Sample**	**Company's workforce**
Gender	Female	26	25
	Male	74	75
Age	≤ 30 years	18	8
	31–40 years	15	16
	41–50 years	25	28
	51–60 years	33	42
	>60 years	10	6
Professional experience	None	11	Not available
	<5 years	7	
	5–10 years	8	
	11–20 years	15	
	21–30 years	34	
	31–40 years	25	
Seniority	≤ 5 years	30	25
	6–10 years	16	13
	11–20 years	20	25
	21–30 years	20	29
	>30 years	15	8
Location	Location 1	43	36
	Location 2	26	28
	Location 3	31	36
Organizational unit	Training and development	14	3
	Insurance systems	15	18
	Central systems	19	20
	Corporate management	8	8
	Order management	19	15
	Customer/Partner service	15	14
	Operations	10	22

### Data Analysis

More than 42 h of interview material were recorded, with interviews lasting between 18 min and 1 h 15 min. After data collection, the interview material was transcribed. In the course of transcription, linguistic details such as pauses in speech, dialect, or rephrasing were smoothed in favor of reading fluency (Mayring, 2014). This procedure is legitimate, as linguistic details did not play a considerable role in answering the research questions (Oliver et al., 2005). To test for objectivity and reliability of the codings, Cohen's Kappa coefficient was calculated by double coding 20% of the data (*N* = 12). Results were above 0.80 suggesting high reliability of the codings (κ influencing factors = 0.83; individual ranking: κ weighted = 0.81, κ unweighted = 0.83; κ commitment = 0.91).

**Figure 2:**
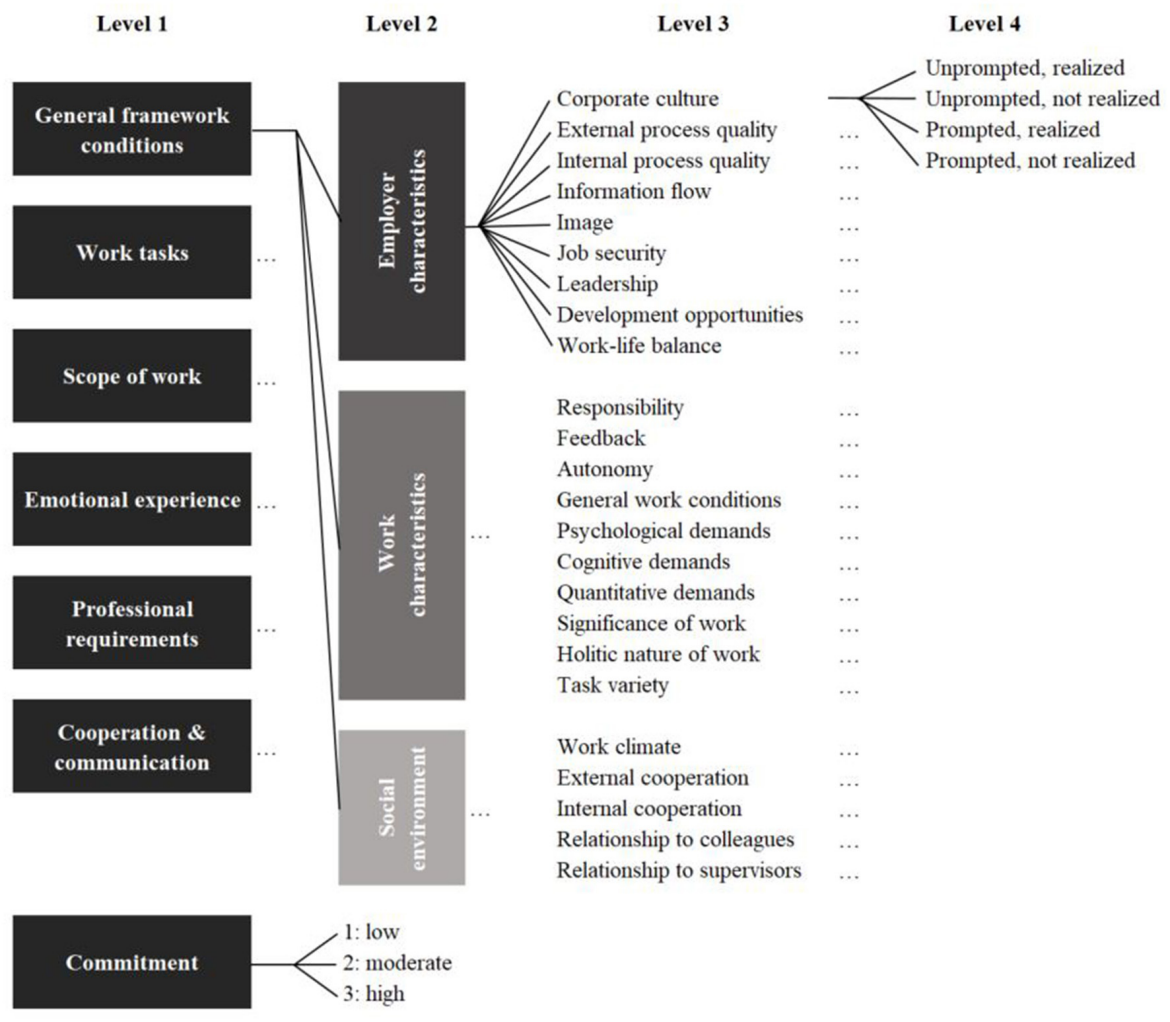
Coding system.

We applied qualitative content analysis (Mayring, 2015) to analyze the data material. Hereby we deductively coded the statements of the participants with a coding system derived from the literature (Figure 2). The coding system contains codes for the six questions on employees' perception of the work environment and the question concerning their commitment. Interview statements addressing the participants' work environment were first assigned to one of the six codes for the respective interview question (level 1) and one of the three categories of conducive factors to well-being at work, namely employer characteristics, work characteristics, or social environment (level 2). This was followed by coding which factor was specifically addressed (level 3). For each influencing factor on level 3, we provided further codes to distinguish whether the factor was identified by the participants themselves (i.e., unprompted statement) or whether the participant referred to an example given in the question; these examples were considered to have been prompted. We argue that unprompted statements point to a potentially higher subjective relevance than those that were prompted by the interviewers. Combined with data on the participant's perception of each influencing factor (realized or not realized), this approach led to four coding possibilities per statement (level 4). Interview statements about participants' commitment were first assigned to the homonymous code for the associated interview question (level 1). Subsequently, the intensity of commitment was assessed using a three-point scale (level 2). For this purpose, the original five-point scale of the KUT questionnaire (Klein et al., 2014) was compressed as follows: The two lowest levels of the scale, “not at all” and “slightly,” were combined to form “1: low.” Here, negative statements such as “I would not recommend the company to my children” (interview 3.08^1^, line 64) were assigned. The two highest scale levels, “quite a bit” and “extremely,” were combined to form “3: high.” Here, agreeing statements such as “I identify with the company. [It] is more than my employer, almost my life” (interview 1.21, line 74) were coded. The medium level remained but was renamed “2: moderate” for consistency in wording.

After encoding the transcribed data with the coding system, the resulting codes were examined in six analysis steps. Step one serves to answer the research question concerning influencing factors of the work environment (RQ1.1). Therefore, the total number of codings per influencing factor was evaluated on the assumption that participants were more likely to address factors that were important to them. Statements that were made several times were critical, as they could potentially distort the ranking. Such a bias could have resulted from influencing factors being addressed in more than one question (e.g., information flow in the question about general framework conditions, cooperation, and communication). To test the data for bias due to mentioning a factor more than once, we adjusted the number of codings per participant for repeated mentions of a factor. In step two, the individual rankings were analyzed. For this purpose, the rankings were considered both unweighted (UR) and weighted (WR). By weighting the ranking, we acknowledged that the participants expressed the perceived relevance of a factor by determining the ranking order. Weighted and unweighted rankings were analyzed for all six questions together and for each question separately. Next, an index was calculated that combined the number of codings with the weighted ranking scores (step three). With this approach, we considered that some factors may have been addressed often without having been ranked by the participant.

To find out how well the participants perceived the influencing factors as having been realized in their daily work environment (RQ1.2), the statements were subdivided into the four coding options resulting from the standardized coding frame (step four). The distinction between realized and not realized aspects provided information about how well the participant perceived them as having been realized. The distinction between prompted and unprompted statements illustrates how important the individual aspects were for the participant (subjective relevance). By combining these four coding options it was possible to derive four recommendations for action about potential innovations in the workplace^2^: Aspects mentioned without a prompt should be *promoted* if they were coded as realized, or require *optimization* when coded as not realized. Aspects referring to a prompt and coded as not realized should be *observed*. Those coded as realized should be *retained*.

Socio-demographic differences (RQ2) were examined in step five of the analysis. Information on gender, age, professional experience, seniority, and type of employment were gathered through the online survey we sent out with the information letter. Analogous to research question one, the number of codes per sociodemographic group and per influencing factor was determined.

To investigate whether there is a link between employees' perception of the work environment and their organizational commitment (RQ3), we calculated a degree of realization for each participant and related it to their statements on commitment. The degree of realization indicates the percentage of a participant's statements in which aspects of the work environment were addressed as positively implemented either by themselves (unprompted) or in response to an example given by the interviewer (promoted) (step six). Hence, the four coding options from analysis step four serve as the basis for calculating the degree of realization.

## Results

Based on the different analysis steps, the following results can be reported from the interview study:

RQ1.1: What aspects of the individual work environment do employees perceive to be conducive to carry out their work tasks?

Concerning the research question concerning factors of the work environment conducive to fulfill one's job duties (RQ1.1), the ranking that resulted from counting the number of codings (step one) gave a first indication of the factors' relevance (Table 2). The results were led by information flow with a total of 200 codings, followed by internal cooperation (195 codings), and work climate (132 codings). Evaluating the number of codings adjusted for the participants resulted in slightly different ranking order. The adjusted number of codings reflects how many participants mentioned an aspect, regardless of how often a participant addressed the respective aspect. Now, internal cooperation and work-life balance ranked first (57 participants mentioned these aspects), followed by information flow (mentioned by 55 participants), work climate, and internal process quality (54 participants each). Nevertheless, the same factors remained in the top three places. The better ranking position of work-life balance could be explained by its functioning as an opening example for the question relating to general framework conditions. Giving examples of specific factors could have caused a so-called priming effect, leading to an overestimation of these factors (Vitale et al., 2008). However, since work-life balance was the only factor for which such a change was observed, a general priming effect can be denied.

**Table 2:** Quantitative analysis of statements sorted by index.

**Influencing factors**	**Number of codings (*N* = 58)^†^**		**Individual ranking (*N* = 61)^‡^**					
	**Total**	**Adjusted for participant**	**Rank 1**	**Rank 2**	**Rank 3**	**Un-weighted^$^**	**Weighted^$^**	**Index^$^**
Internal cooperation	195	57	66	56	15	137	325	520
Information flow	200	55	28	66	19	113	235	435
Work climate	132	54	46	39	15	100	231	363
Professional development	93	52	17	34	17	68	136	229
Leadership	109	49	13	28	20	61	115	224
Internal process quality	123	54	8	27	16	51	94	217
Work-life balance	96	57	25	14	11	50	114	210
Feedback	91	48	16	29	9	54	115	206
Autonomy	74	46	19	17	12	48	103	177
Quantitative demands	78	50	14	19	10	43	90	168
Working conditions	94	49	6	20	14	40	72	166
Relationship to colleagues	72	48	15	21	7	43	94	166
Cognitive demands	66	41	18	16	5	39	91	157
Psychological demands	73	43	7	13	7	27	54	127
Variety	49	42	13	15	3	31	72	121
Significance	57	47	8	5	14	27	48	105
Corporate culture	52	39	6	4	8	18	34	86
Responsibility	33	33	7	13	5	25	52	85
Relationship to supervisor	44	33	1	5	1	7	14	58
Holistic nature	41	36	3	3	2	8	17	58
Image	45	39	1	1	3	5	8	53
Job security	29	28	4	3	1	8	19	48
External process quality	21	16	2	4	2	8	16	37
External cooperation	14	13	0	2	1	3	5	19
Total:	1,881	–	–	–	–	–	–	–

† *58 of 61 participants gave permission for tape recording*.

‡ *Inclusion of the entire sample is possible as the ranking results were also recorded in handwriting*.

$ Exemplary calculations for internal cooperation: unweighted ranking: (66 + 56 + 15) = 137; weighted ranking: (3 * 66 + 2 *56 + 1 *15) = 325; index: 195 + (3 *66 + 2 *56 + 1 *15) = 520.

Evaluating the perceived relevance of influencing factors using the individual rankings (step two) showed a comparable result (Table 2). This applied to the analysis of both the unweighted ranking (UR) and the weighted ranking (WR). For both assessments, it could be observed that internal cooperation now came first (UR = 137, WR = 325), information flow second (UR = 113, WR = 235), and work climate third (UR = 100, WR = 231). However, considering the rankings for each of the six questions separately, other factors achieved higher rankings for individual questions. Nevertheless, an aggregated view of the rankings seemed appropriate, since no systematic pattern could be identified, and our research was focused on the evaluation of the work environment as a whole. Finally, calculation and analysis of the index (step three) resulted in the same three factors on top, led by internal cooperation (520 points), followed by information flow (435 points), and work climate (363 points). Taken all together, RQ1.1 can be answered as follows: Employees perceive *internal cooperation, information flow*, and *work climate* as the three most conducive factors for fulfilling their work tasks.

RQ1.2: To what extent perceive employees specific factors of the work environment as realized in their daily work context?

**Figure 3:**
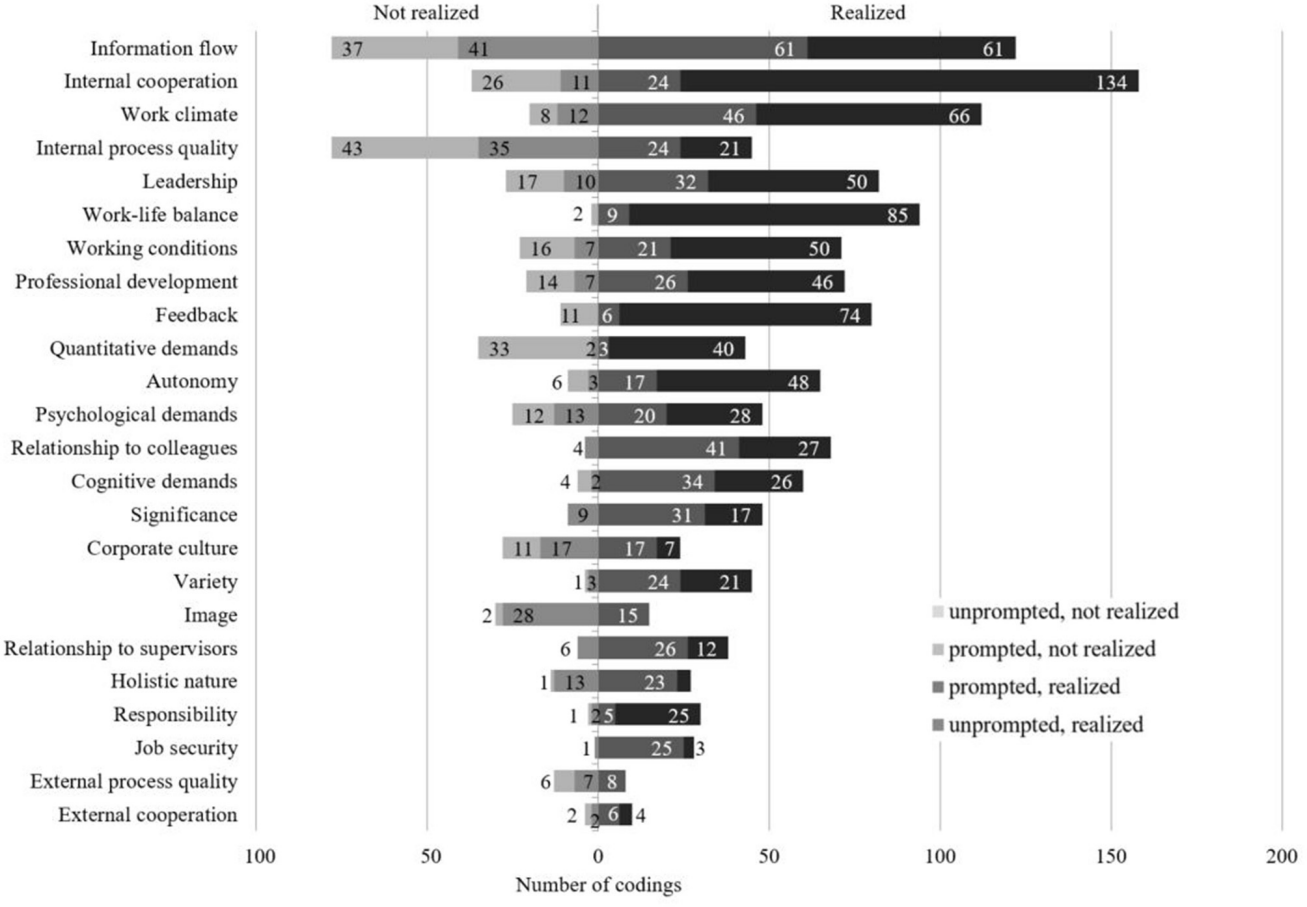
Quantitative analysis of subjective relevance and perceived implementation per influencing factor.

To answer the research question on how the factors are experienced in the daily work context (RQ1.2), the four possible coding options were considered first (step four). Depending on the perceived realization of a presage factor as well as its subjective relevance for the participant, a statement could be coded either as unprompted-not realized, prompted-not realized, prompted-realized, or unprompted-realized. Figure 3 shows that the realization of most factors was considered to be positive: The majority of the statements were perceived as having been satisfactorily realized. In addition, a large proportion of them was mentioned without prompt (see e.g., internal cooperation, work climate, work-life balance, and feedback). This shows the comparatively high importance of these factors for the participants. At the same time, some aspects were considered to not have been realized satisfactorily. Here, too, statements without prompt had greater subjective relevance. It is noticeable that about one-third of the statements concerning the organization's internal process quality were assigned to this code category (43 of 123 statements). The score for quantitative demands was 42 %. In summary, it can be stated for RQ1.2 that the participants assessed the realization as satisfactory for the majority of the presage factors. Nevertheless, participants identified strengths and deficits for the same factors.

**Figure 4:**
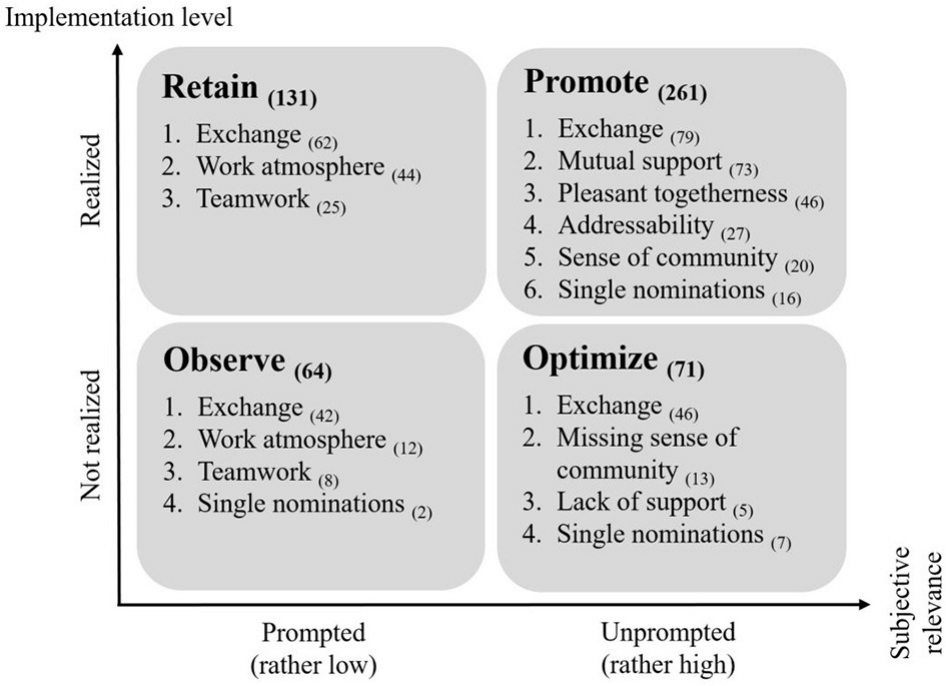
Recommended actions for aspects of internal cooperation, information flow, and work climate, sorted by number of codings.

To shed more light on the specification of presage factors discussed by the IT professionals, we classified the statements on the three most relevant factors—internal cooperation, information flow, and work climate—into overarching categories (e.g., exchange, work atmosphere, teamwork, see Figure 4). Subsequently, each category was assigned to one of four recommendations for action—namely retain, promote, observe, or optimize—according to its subjective relevance and perceived implementation level. Based on these recommendations for action, suggestions for innovations in the workplace can be derived that take into account the needs of the interviewed IT professionals as well as the perceived workplace conditions per topic category. It should be noted that a category could potentially be assigned to several recommendations for action. This is because several aspects were summarized under one category (e.g., information transfer and contact persons are both aspects of the category exchange). In addition, different participants might have considered the same aspect differently well-implemented or differently relevant, resulting in different recommendations for action for the respective aspect.

For example, the category *exchange* was assigned to each of the four recommendations for action. Some employees valued, and frequently mentioned, intra- and inter-divisional knowledge sharing, which should be promoted accordingly. Regarding intra-divisional knowledge sharing, one IT professional reported that knowledge within the team is actively shared by its members so that the team's functionality is guaranteed at all times: “In our unit, everyone knows everything and can replace every one. We don't have anyone who is completely isolated with a specialized area of expertise” (interview 1.03, line 68). Also lauded were the exchange of experience, prioritization of tasks, availability of information, and documentation of knowledge in an always-accessible repository. In this context, another professional emphasized that the team members exchange information, especially regarding problematic issues: “We talk to each other within the department—where problems arise, where developments do not run properly” (interview 1.26, line 61). In contrast, others criticized intra- and inter-divisional knowledge sharing due to heterogeneous knowledge levels, outdated or delayed information, poor transparency, and prioritization, as well as an insufficient information flow (optimize). According to one employee, job duties are often assigned at short notice, leaving little time for adequate preparation and processing: “Most of the time, things are put in front of you that you have nothing to do with and that usually come very spontaneously and are best completed by the day after tomorrow. So of course I don't have time to get exactly into it [and prepare] thoroughly” (interview 3.02, lines 145–147). For some, exchange was less important. They only addressed the above-mentioned aspects in response to questions from the interviewer. For instance, the availability of information was deemed satisfactory (retain): “Well, I think you always have the information you need for your job. Some things would be nice to know, but it doesn't affect my work” (interview 1.23, line 32). The quantity of information, however, was criticized, but at the same time, the concerned employee explained that they had come to terms with the abundance of information material: “The information flow is there, but sometimes too much. I don't always need to be on the mailing list if it's none of my business. [.] 80 percent of my colleagues don't read it either. I know what I have to do and if I don't, I get the information elsewhere” (interview 1.03, lines 53–57) (observe).

Work atmosphere and teamwork were also mainly discussed in response to questions from the interviewer. One professional, for example, described a positive *work atmosphere* characterized by a harmonious and collegial climate between the team members: “The work atmosphere with us and in our environment [.] is very good. And it's a pleasure to work when you know that your colleagues are able or willing to help you if you have any problems. And you don't have to beg, but one shout is enough and there are three people ready to do something for you” (interview 2.06, line 7; retain). Worth observing are instead tensions in the team and a lack of a sense of community, which other professionals criticized. Similarly, with *teamwork*, some participants reported departmental differences and needs for improvement on the operational level (observe), while others had a positive perception of teamwork, praising the reliability of their colleagues, and their constructive ways of working (retain).

However, two facets of work atmosphere and teamwork also appeared in the recommendation option optimize. On the one hand, this concerned a *missing sense of community*. Participants reported on competitive and hierarchical thinking, a lack of mutual understanding, and lines of demarcation between the company's three locations. For one employee, this becomes particularly clear when working across divisions: “[then] our sense of community is limited and I'm not always sure whether we're all pulling in the same direction. Individual interests come to the fore and, if something doesn't work, people try to find someone outside their ranks to blame” (interview 1.23, line 58). On the other hand, some mentioned a lack of support in solving work-related problems. Either because “people don't help you as much to get ahead themselves” (interview 2.12, line 6). Or, because there is simply no other employee who is familiar with the respective topics: “I am alone with my area and I have to find a solution alone. I also don't have a representative” (interview 3.08, line 43).

Despite these improvement needs, half of the statements were assigned to the recommended action promotion. This proved a satisfactory implementation of the presage factors with high subjective relevance for the sample. One topic most participants rated as positive was *mutual support*. They appreciated good coordination of tasks, professional support from colleagues and supervisors, and constructive discussions, and cooperation to increase productivity. An employee described the collaboration as follows: “What I find very beneficial is the collegial behavior. […] You discuss things very openly and directly, but it's never personal. I find that very conducive to the work atmosphere and of course that also has an impact on our output when the team harmonizes well” (interview 1.04, line 6). This statement also illustrates the *pleasant togetherness* that results, among other things, from respectful and familial interaction. Moreover, *the addressability of colleagues and supervisors* seemed to be important for some participants. They emphasized that “in terms of addressability, there is always someone there” (interview 1.20, line 53) and “conversations [are] also possible across hierarchies” (interview 1.26, line 8). Overall, the prevailing *sense of community* scored highly, allowing participants to perceive their work environment as a place of common goals and interests.

RQ2: Does employees' perception of factors of the work environment differ for distinct sociodemographic groups?

The research question on sociodemographic differences (RQ2) was examined by evaluating the number of codings per polled sociodemographic factor (step five). Results showed that the factors internal cooperation and information flow occupy the first two places in almost all sociodemographic groups (see Table 3). Here, it is noticeable that internal cooperation was ranked higher by female IT professionals and older employees. The picture is reversed for information flow, which was considered more important by male IT professionals and younger employees. Another point worth mentioning is that employees with long seniority value internal process quality more highly than all other groups. The third most important factor was work climate; ranked on position three by nine out of 20 groups. In the other groups the factors internal process quality (9x), leadership (3x), working conditions (1x), feedback (1x), internal cooperation (1x), and professional development (1x) ranked third, revealing a more mixed picture compared to the first two ranking positions. Overall, the influence of sociodemographic factors can be classified as rather low for our sample, despite the fluctuations described.

**Table 3:** Influencing factors' ranking positions (upper two quartiles, sorted by sociodemographic factor).

**Sociodemographic factor**	**Gender**		**Age (in years)**					**Professional experience (in years)**						**Seniority (in years)**					**In training and development**	
**Specification (No. of participants)**	**Female (16)**	**Male (45)**	**≤30 (11)**	**31–40 (9)**	**41–50 (15)**	**51–60 (20)**	**> 60 (6)**	**None (7)**	**<5** **(4)**	**5–10 (5)**	**11–20 (9)**	**21–30 (21)**	**31–40 (15)**	**≤5 (17)**	**6–10 (11)**	**11–20 (12)**	**21–30 (12)**	**>30 (9)**	**Yes (8)**	**No (53)**
**Influencing factors (sorted by index)**																				
Internal cooperation	**1**	2	2	2	2	**1**	**1**	2	2	**1**	**1**	2	**1**	2	**1**	**1**	**1**	3	2	**1**
Information flow	2	**1**	**1**	**1**	**1**	2	2	**1**	**1**	2	**1**	**1**	2	**1**	**1**	2	2	**1**	**1**	2
Work climate		3	3					2	3			3		3	3	3			3	
Professional development								3												
Leadership				3					3	3	2				2					
Internal process quality	3			3		3	3			3	3		3			3		2		3
Work-life balance																				
Feedback					3															
Autonomy																				
Quantitative demands																				
Working conditions																	3			
Relationship to colleagues																				

*Ranks were placed twice if more than one factor scored equal index values. Rank position 1 are indicated in bold*.

RQ3: Is there a relationship between employees' perception of the work environment and their commitment to the employer?

Regarding perceptions of the work environment, the results for RQ1.2 are mirrored, as shown by the average degree of realization of 75.2% (*SD* = 12.7). Specifically, more than two-thirds of the interviewed IT professionals perceived their work environment as satisfactory. Another nine IT professionals even experienced their workplace as particularly positive. Concerning commitment, it is noticeable that a predominantly positive assessment was also made here (*M* = 2.72; *SD* = 0.073). This seems understandable, as organizational commitment and turnover intention correlates negatively (Koslowsky et al., 2012; Agarwal and Sajid, 2017). Thus, employees who feel little commitment to their employer are more likely to switch. Accordingly, employees with high commitment remain.

**Figure 5:**
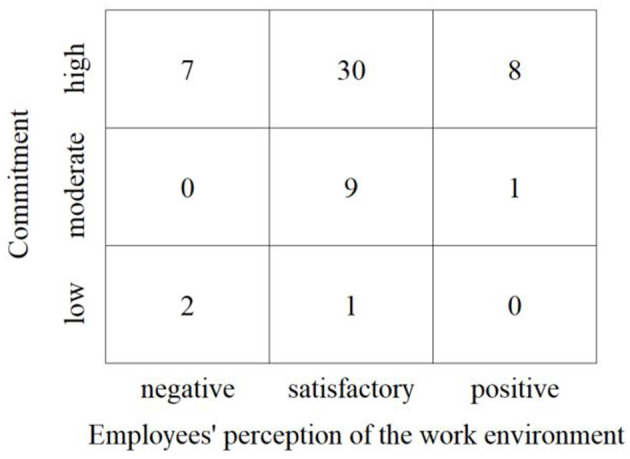
Perception matrix.

Findings on the link between employees' perceptions of the work environment and their commitment (RQ3) are shown in Figure 5. The horizontal axis depicts employees' perception of the work environment. The vertical axis displays their commitment. Regarding employees' perception of the work environment, we distinguished three groups—negative, satisfactory, and positive. This allowed clustering of the participants and subsequent comparison of the groups. To build the three groups, a standard deviation (12.7) was subtracted from the average degree of realization (75.2%) and added, respectively. Employees with a degree of realization below 65.52% were thus assigned to the group with a negative perception, employees with a degree of realization between 65.52 and 87.92% to the group with satisfactory perception, and employees with a degree of realization above 87.92% to the group with a positive perception. In combination with the statements on commitment (low, medium, high), this resulted in a matrix of nine fields showing the extent to which the perception of the work environment and perceived organizational commitment are linked. Based on their statements concerning the work environment and their commitment, the interviewees were located within this nine-field matrix.

There are indications of a positive relationship between the perception of the work environment and individual commitment. Although only a weak and non-significant relationship could be found between the IT professionals' commitment and their degree of realization (Spearman's Rho = 0.089), a Kruskal-Wallis test showed that the degree of realization differed significantly across the three commitment levels (Kruskal-Wallis-H = 7.317, *p* = 0.026). Subsequent *post-hoc* tests (Dunn-Bonferroni tests) revealed significant differences in the degree of realization between participants with low and medium commitment (*z* = −2.676, *p* = 0.007, *r* = 0.74) and participants with low and high commitment (*z* = −2.480, *p* = 0.013, *r* = 0.36). Participants who rated their work environment positively also rated their commitment to their employer positively. This applies to eight IT professionals. Based on the two participants who rated their work environment most positively (degree of realization each about 96%), it can be exemplified where this particularly good assessment stems from. On the one hand, they were satisfied with the factors of their work environment to which they attached particular importance in the performance of their work. On the other hand, they also had a positive perception of the other factors that play a role in their day-to-day work. For a comparably large group of 30 IT professionals, a satisfactory perception of the work environment was observed with the same high level of commitment. The two participants whose assessment of the work environment was closest to the mean (degree of realization 74 and 77%, respectively) were noticeably more deficient than the participants in the first group (positive perception and high commitment). And this applied both to the factors that are most important to them and the rest of the factors shaping their work environment. In particular, the two participants expressed potential for optimization for the factors to which they attached the greatest importance in their day-to-day work. Conversely, it was found that participants who criticized some aspects of their work environment (negative perception) also indicated low commitment. The more the degree of realization decreased, the more likely factors were rated as unsatisfactory. The two participants with the lowest degree of realization (each about 49%) reported, for example, that aspects of their work environment that were particularly important to them were inadequately realized. Moreover, they were also not satisfied with other factors that make up their work environment. A surprising result was shown by the seven participants who indicated a high level of commitment despite a rather negative perception of their work environment. Comparable to the participants with negative perception and low commitment (lower left box), they criticized the factors of their work environment that are particularly important to them in everyday working life. At the same time, they identified optimization potential for other factors, whereby a mixed assessment was observed here, i.e., both negative and positive statements were made.

Concerning RQ3, it can be stated that there seems to be a positive relationship between employees' perception of the work environment and their commitment. The perception of the overall work environment appears to be decisive, especially if, from the employee's point of view, those factors that are particularly important to them in their everyday work are not implemented satisfactorily. Consequently, if an employer succeeds in designing the work environment in such a way that employees perceive it as conducive to the performance of their work, this favors the relationship between employees and employer and promotes long-term employment relationships. Nevertheless, results also indicate that there may be other factors besides the perception of the work environment that lead to high commitment.

## Discussion

Taken together, the findings of the interview study show that—from an employee perspective—three of the well-being-promoting factors have proven to be especially relevant. Employees experience particularly interpersonal relationships as conducive to fulfilling their work tasks. Furthermore, interpersonal relationships are crucially important in respect of how one assesses the individual work environment: First, they contribute to an environment in which employees feel safe and affiliated. This may lead to a comfortable state in which each of the parties concerned feels valued and indispensable. Second, these interpersonal relationships are the basis of an organization-wide network. This is of particular importance concerning knowledge acquisition and skill development. Results also show that there can be differences in the perception and implementation of certain influencing factors, making one-fits-all solutions not very promising. Moreover, employees seem to benefit most from informal learning opportunities, such as sharing experiences or receiving ad hoc support from colleagues, for performing everyday work tasks. Finally, employees' perceptions of the work environment appear to have a positive relationship with their commitment, whereby the perceived realization of the individually most relevant influencing factors seems to play a crucial role.

When interpreting our findings, however, the limitations of the study must also be taken into account. First, the subjective relevance of specific factors for the assessment of the work context may vary over time. Changes in the relevance of single factors would possibly be reflected in changes in well-being at work. This may be caused by changes in the work environment, such as new tasks and colleagues, or changes in other areas of life, such as a new family constellation or a new place of residence. Future research projects should therefore be designed as panel studies to investigate whether the identified influencing factors are constant in the long run. This could help to differentiate between stable and variable factors conducive to well-being at work.

Second, qualitative research projects have limitations that are inherent in the method and provide starting points for complementary quantitative research efforts. For instance, a questionnaire study could be developed from the present results, containing scales on the influencing factors (independent variables), their perception (possibly moderator or mediator variables) and the outcome variables well-being at work and commitment. In this way, the relationship between the perception of the work environment and commitment could be investigated in more detail. It would also be interesting to see whether the factors considered being conducive to well-being at work compensate for deficits in other factors. For example, high quantitative requirements or unfavorable internal processes could be compensated by the support of colleagues. Moreover, the influence of personality traits could be investigated. These were not considered in our study. However, some evidence suggests that a positive evaluation and satisfaction with the tasks can only develop if the requirements match the personality characteristics of the employee (Christiansen et al., 2014). For example, playful characters and employees who are open to experience can benefit from the independent design of their work tasks. Integrating fun and competition into daily tasks can increase their creativity and commitment (Scharp et al., 2019). In addition, agentic employees tend to adapt their work environment to their individual needs and expectations (Goller, 2017).

Third, the study design's focus was on the individual employee. This does not take into account that the organizational work context requires a great deal of interaction, and thus employees hardly act in isolation. The results support this assumption in that they prove the importance of interpersonal interactions. Thus, group discussions could serve to offer deeper insights concerning the dynamics of social interactions within the work context (Krueger, 1999). Therefore, it would be interesting to further analyze organizational units to investigate how their daily work is organized. Studies of this type could also help to elucidate the relationship between well-being and performance at the group level for which evidence has been scarce. More research efforts are therefore needed that can shed light on the causality and reciprocity between the two variables (García-Buades et al., 2020). It would also be interesting to compare the extent to which employees' perception corresponds with the perception of the employer. Differences in the perception of the work environment could hinder effective interventions to improve working conditions. Hence, future studies should survey the perception of the employer in addition to the individual perception of the employees.

Fourth, the generalizability of our results is limited, as organizational and industry characteristics may have influenced employees' perception of the work environment. Therefore, the results require validation through studies in other organizations and industries. Furthermore, it is possible that selection effects arising from sample recruitment, e.g., through overrepresentation of particularly satisfied and committed employees, could have affected the findings. The same would apply to the possibility of socially desirable response patterns and the avoidance of specific sensitive issues, such as relationships with supervisors. However, as the results show a quite differentiated evaluation of the work environment, concerns about selection effects and social desirability can be discounted. And finally, it has to take into account that we only report data from one company with specific characteristics concerning the profession, age structure, and gender. This is due to the rather difficult conditions of field access. This further reduces the generalizability of the results. It is therefore essential to follow up with further studies in other companies to corroborate the results reported here.

Overall, the findings of our research are in line with other research showing that employees perceive positive relationships with colleagues and support from the team and supervisors as particularly useful and helpful to cope better with challenging phases (Alegre et al., 2016; Van der Heijden et al., 2020). In such contexts, employees are more committed, and at the same time their willingness to learn and exchange increases (Zboralski, 2009; Huang et al., 2016; Frazier et al., 2017). To strengthen interpersonal relationships, organizations could implement team-building activities. This would address mutual trust and reliability. In addition, information on the responsibilities and competencies of colleagues would help to find appropriate contact persons and to build up a professional network. For implementing knowledge sharing as part of the daily work routine, employers should create appropriate conditions on the organizational level, such as providing the necessary tools and resources (Lancaster and Di Milia, 2014), and anchor knowledge sharing and teamwork in the organization's corporate culture (Jeong et al., 2018).

Particular emphasis should be placed on encouraging informal learning opportunities, such as sharing experiences or *ad hoc* peer support, as employees benefit most from these practices, according to our study. Discussing best practices and lessons learned in meetings at the team or department level could also be a viable path in this context. Employers should also offer retreats for undisturbed exchange between employees. To communicate experiences across departments and locations, they could be published in a tweet-like format on the organization's intranet. Overall, increased communication of individual experiences would boost employees' visibility and convey a feeling of appreciation. This should be taken into account when designing the work environment, e.g., by using flexible communication tools and providing time capacities for mutual exchange. At the group level, feedback, internal and external networking, and the quality of interpersonal relationships are all crucial (Schürmann and Beausaert, 2016; Jeong et al., 2018). These aspects at the group level have been identified as largely implemented, which indicates a supportive work environment at least in some parts of the company (best practice). Such concrete indications for designing a work environment in which employees feel socially embedded can help to boost sustainable well-being at work.

At the same time, our findings highlight the need for employers to examine how employees experience and perceive implemented measures to ensure that beneficial factors, such as communication tools or training opportunities, have positive effects on employees' well-being at work. To this end, employees' perception should be evaluated regularly. In teams in which the exchange is already functioning well, evaluations can be carried out as needed and bilaterally. In teams in which the exchange has not worked well so far, evaluation should be introduced based on predefined evaluation questions and with predefined appointments (e.g., in annual reviews or team meetings). A regular evaluation of the perception of the work environment could—as the results show—help to identify unfavorable developments and initiate appropriate countermeasures to design a well-being-friendly work environment. In case the work environment is not yet optimally designed from the employees' perspective, job crafting interventions can be helpful. Through systematic training, employers can show their employees how to make self-directed and targeted changes to the resources and requirements of their work environment (Van Wingerden et al., 2017). If employees succeed in adapting work demands according to their individual needs (job crafting), they benefit from more professional development opportunities as well as increased self-efficacy, better performance, and enhanced well-being (Van Wingerden et al., 2017).

Overall, this organizational context shows the characteristics of an expansive work environment. Such environments enable employees to exchange knowledge and experience, acquire new knowledge, and further their skill development. Moreover, an appreciative and innovative atmosphere is typical of an expansive work environment (Fuller and Unwin, 2004). In summary, our approach has provided a detailed overview of workplace conditions that can influence employees' professional development and potentially impact their well-being at work. Furthermore, we identified which specific aspects of the work environment can induce positive perceptions of the work environment. Results show that IT professionals especially perceive interpersonal relationships in a positive way. If they experience these as positive, employees benefit from a good flow of information, good internal cooperation, and a pleasant work atmosphere in performing their everyday work tasks. The in-depth analysis of employees' statements helped to determine which aspects of the work environment should be implemented, developed or promoted. In the long run, this can support individual learning and development paths and generate a work environment that sustainably promotes employees' well-being at work. Thus, employers can respond to employees' needs by analyzing the subjective significance of certain influencing factors and uncovering the potential for their implementation.

## Data Availability Statement

The datasets presented in this article are not readily available because making the generated datasets available requires the agreement of the cooperating institution from which the data were collected. Requests to access the datasets should be directed to wipaed2@mail.uni-mannheim.de.

## Ethics Statement

Ethical review and approval was not required for the study on human participants in accordance with the local legislation and institutional requirements. The patients/participants provided their written informed consent to participate in this study.

## Author Contributions

SZ and JS contributed equally to the conception and theoretical development of this work and to the elaboration of the models used. SZ has collected the data on which this work is based. She was supported by student assistants. All authors have seen and approved the final version of the manuscript.

## Conflict of Interest

The authors declare that the research was conducted in the absence of any commercial or financial relationships that could be construed as a potential conflict of interest.

## Publisher's Note

All claims expressed in this article are solely those of the authors and do not necessarily represent those of their affiliated organizations, or those of the publisher, the editors and the reviewers. Any product that may be evaluated in this article, or claim that may be made by its manufacturer, is not guaranteed or endorsed by the publisher.

## Footnotes

^1^All interviews were named with a code. The number before the dot indicates the location where the interview was conducted. The two numbers after the dot indicate the consecutive interview number, e.g. 3.08 for interview 8 at location 3.

^2^The distinction in four recommendations for actions closely resembles Henderson's BCG-matrix (1979)—a widely used instrument for strategic product management. For this purpose, products are classified into the matrix according to market share and market growth, from which recommendations for further sales can be derived.
